# Why Users Rebel Against Algorithms: The Impact of Perceived Algorithmic Power on Fairness Evaluations, Negative Emotions, and Resistance Behaviors

**DOI:** 10.3390/bs16071044

**Published:** 2026-06-23

**Authors:** Yangyang Shi, Jialu Wang, Jing Chen, Haiqing Bai

**Affiliations:** 1 School of Journalism and Communication, Xiamen University, Xiamen 361005, China; 31920230157320@stu.xmu.edu.cn (Y.S.); 13345236380@163.com (J.C.); 2 School of Arts, Design & Architecture, University of New South Wales, Sydney, NSW 2052, Australia; 720966wjl@gmail.com

**Keywords:** algorithmic resistance, algorithmic power, algorithmic technicality, fairness evaluations, negative emotions

## Abstract

Platform algorithms are widely used to personalize content and organize users’ everyday social media experiences. Yet they may also become objects of resistance when algorithmic recommendations are perceived as intrusive, repetitive, or difficult to escape. Drawing on the critical theory of technology, this study develops a parallel mediation model to explain why users resist algorithm-driven social media platforms. Focusing on algorithmic power and algorithmic technicality as two perceived characteristics of platform algorithms, the model examines whether these perceptions are associated with algorithmic resistance through fairness evaluations and negative emotions. Based on survey data from users of Chinese algorithm-driven social media platforms, the results show that both algorithmic power and algorithmic technicality are associated with stronger algorithmic resistance through lower fairness evaluations and stronger negative emotions. These findings suggest that algorithmic resistance is not merely a response to inaccurate or opaque recommendations, but also reflects users’ reactions to algorithms experienced as systems of platform control and data-driven inference. By identifying fairness evaluations and negative emotions as parallel cognitive and affective pathways, this study shifts attention from algorithmic acceptance to algorithmic resistance and provides a more critical understanding of user agency in human–algorithm relations.

## 1. Introduction

Algorithm-driven social media platforms have become deeply embedded in everyday digital life. Through personalized recommendation, content curation, and behavioral prediction, platform algorithms shape what users see, how they interact, and which choices become more visible or convenient (Berman & Katona, 2020). Across platforms such as WeChat, Weibo, Xiaohongshu, Douyin, and TikTok, algorithmic personalization has become a central mechanism through which social media environments are organized and experienced (Eg et al., 2023). Although these systems can improve access to relevant content, they also make users’ platform experiences increasingly shaped by algorithmic classification, ranking, and prediction. When embedded in platform logics of attention and engagement, algorithms may reinforce prior preferences, narrow exposure to alternative content, and draw users into repetitive or homogeneous information environments (Bakshy et al., 2015; Flaxman et al., 2016). Prior research has linked these dynamics to filter bubbles, information cocoons, algorithmic fatigue, and declining digital well-being (Ahmmad et al., 2025; Balaskas et al., 2025; Liu et al., 2025).

As these experiences accumulate, users do not simply remain passive recipients of algorithmic influence. They may develop everyday forms of algorithmic resistance, such as clicking “not interested,” modifying interaction signals, avoiding recommended content, or reducing platform use. Prior work has documented varied practices of algorithmic resistance, including avoidance, obfuscation, productive adjustment, disengagement, and contestation (Ettlinger, 2018; Velkova & Kaun, 2021; Xie et al., 2022). Recent work further conceptualizes algorithmic resistance as an everyday practice through which users seek agency in response to platform power (Bonini & Treré, 2024; Velkova & Kaun, 2021). Accordingly, this study examines algorithmic resistance as an overall behavioral tendency through which users seek to limit, redirect, or challenge algorithmic influence, while recognizing that such resistance may take varied everyday forms.

Despite the growing relevance of algorithmic resistance, our understanding of why users resist platform algorithms remains limited. Prior research has examined algorithmic opacity, privacy concerns, declining trust, algorithmic fatigue, and avoidance behavior in personalized digital environments (Lina & Setiyanto, 2021; Shin, 2020; Tayeb et al., 2024), while qualitative and interpretive studies have shown how users make sense of algorithms, develop folk theories, and tactically adjust their behavior in response to algorithmic systems (Bishop, 2019; Bucher, 2017; Karizat et al., 2021; Siles et al., 2024). However, less is known about which perceived characteristics of platform algorithms lead users to resist and through what cognitive and affective mechanisms these perceptions are associated with resistance-related behaviors, particularly users’ fairness evaluations and negative emotions toward algorithmic intervention.

To address this gap, this study develops and tests a parallel mediation model of algorithmic resistance by drawing on the critical theory of technology (Feenberg, 1991, 1999). The model distinguishes algorithmic power, which refers to users’ perceptions of algorithmic control, visibility allocation, rewards, penalties, and constraints (Bucher, 2012; Kellogg et al., 2020), from algorithmic technicality, which refers to users’ perceptions of data-driven tracking, inference, and prediction (Adomavicius & Tuzhilin, 2005; Gillespie, 2014). Using online survey data from algorithm-experienced users of Chinese social media platforms, this study measured algorithmic power, algorithmic technicality, fairness evaluations, negative emotions, and algorithmic resistance with multi-item survey scales. The results show that both algorithmic power and algorithmic technicality are associated with stronger algorithmic resistance through lower fairness evaluations and stronger negative emotions. These findings suggest that users resist algorithms not simply because recommendations are inaccurate or opaque, but because algorithmic systems may be evaluated as unfair and experienced as intrusive or unsettling (Lee, 2018; Shin, 2020).

By focusing on the psychological mechanisms of algorithmic resistance, this study contributes to platform algorithm research in three ways. First, it shifts attention from algorithmic acceptance to algorithmic resistance, highlighting how users respond when personalization is experienced as constraint rather than convenience. Second, it distinguishes algorithmic power and algorithmic technicality as two perceived characteristics of platform algorithms. Third, it identifies fairness evaluations and negative emotions as cognitive and affective pathways linking perceived algorithmic characteristics to resistance-related behaviors. Together, these contributions offer a more critical account of user responses to algorithmic influence in everyday social media environments, with evidence from Chinese algorithm-driven platform contexts.

## 2. Theoretical Foundation and Research Hypotheses

### 2.1. Critical Theory of Technology

This study draws on the critical theory of technology to conceptualize platform algorithms as socio-technical systems rather than neutral instruments of efficiency. Platform algorithms are often understood as technical tools that improve recommendation efficiency and optimize user experience. Such an instrumental view, however, is insufficient for explaining why users may question, avoid, or resist algorithms in everyday platform use. The critical theory of technology suggests that technologies are embedded in social arrangements, institutional logics, and power relations, and that they shape the conditions under which users act, evaluate, and respond (Feenberg, 1991, 1999). From this perspective, platform algorithms should not be understood only as tools that match users with relevant content, but also as systems that organize visibility, distribute opportunities, guide interaction, and define the conditions of platform participation.

In platform environments, algorithms do not merely filter and recommend content; they also structure visibility, opportunity, feedback, and choice architectures. Prior research similarly shows that algorithms exercise social power by organizing visibility and shaping the conditions of action in digital platforms (Beer, 2009, 2017; Bucher, 2012; Gillespie, 2014). Research on personalized user experiences in social media further shows that algorithmic personalization is closely tied to users’ algorithmic awareness, customization practices, interactions with curated content, and concerns over closed information environments (Eg et al., 2023). Platform algorithms are therefore not merely intermediaries between users and information; they also operate as governance mechanisms that structure users’ behavioral environments and shape how users interpret their relationship with platforms.

Building on this theoretical foundation, the following sections examine algorithmic power and algorithmic technicality as two perceived characteristics of platform algorithms and explain how they may be associated with algorithmic resistance through two parallel mechanisms: fairness evaluations and negative emotions.

### 2.2. Algorithmic Power, Algorithmic Technicality, and Algorithmic Resistance

Building on the critical theory of technology, this study distinguishes between algorithmic power and algorithmic technicality as two perceived characteristics of platform algorithms. Algorithmic power captures the governance dimension of platform algorithms, referring to users’ perceptions that algorithms allocate visibility, regulate opportunities, reward or penalize behavior, and constrain action within platform environments (Alizadeh et al., 2023; Bucher, 2012; Lundahl, 2022). Recent research on platform work also suggests that perceived algorithmic control may have a double-edged effect, functioning not only as an enabling mechanism but also as a source of constraint (Zhu et al., 2026). Algorithmic technicality captures the data-driven dimension of platform algorithms, referring to users’ perceptions that algorithms track behavioral traces, infer preferences, and predict future actions for personalized recommendations (Adomavicius & Tuzhilin, 2005; Gillespie, 2014). This distinction is theoretically important because algorithmic power emphasizes the governance logic of platform algorithms, whereas algorithmic technicality emphasizes their data-driven inferential logic. Together, they capture two ways in which users may experience platform algorithms as consequential in everyday social media use.

Algorithmic resistance refers to users’ everyday behavioral tendency to reduce, avoid, redirect, or counteract the influence of platform algorithms (Bonini & Treré, 2024; Velkova & Kaun, 2021). It does not necessarily involve explicit protest, complete platform exit, or outright rejection of technology; rather, it often appears in low-intensity but intentional practices, such as ignoring algorithmic prompts, questioning recommendations, bypassing algorithmic suggestions, modifying interaction signals, or reducing platform use. Scott’s (1985) notion of everyday resistance helps explain these dispersed practices as meaningful expressions of agency rather than as trivial deviations from normal use. In algorithmic platform contexts, users may also develop folk theories about how algorithms work and use these understandings to negotiate with or resist algorithmic systems (Karizat et al., 2021). Accordingly, this study examines algorithmic resistance as an overall behavioral tendency and focuses on the cognitive and affective pathways through which perceived algorithmic characteristics are associated with resistance-related behaviors.

### 2.3. The Mediating Role of Fairness Evaluations

Fairness evaluations refer to users’ cognitive judgments about whether algorithmic operations are fair, legitimate, consistent, and unbiased. In platform algorithm contexts, fairness is not merely about satisfaction with recommendation outcomes, but a standard through which users assess whether algorithmic influence is acceptable. Prior research links fairness to procedural legitimacy, transparency, consistency, explainability, and bias (Binns et al., 2018; Lind & Tyler, 1988; Shin, 2020). Recent work on recommender systems further emphasizes that fairness is not only a technical issue but also a contextual judgment involving value trade-offs among different stakeholders (Deldjoo et al., 2024), while perceived algorithmic unfairness may become a basis for negative user responses (Sun et al., 2025). For social media users, fairness evaluations therefore concern whether the rules, data practices, and decision processes behind algorithmic recommendations are perceived as reasonable, legitimate, and sufficiently explainable.

Algorithmic power may weaken users’ fairness evaluations because platform algorithms allocate visibility and behavioral opportunities through recommendation, ranking, filtering, rewards, and penalties. These mechanisms can reproduce power asymmetries between algorithmic systems and the users subject to them (Barati & Ansari, 2022; Lundahl, 2022). When users perceive that algorithms prioritize certain content, restrict particular actions, or distribute visibility through opaque rules, algorithmic power may be experienced as platform governance rather than neutral personalization (Beer, 2017). Under such conditions, users may question the fairness and legitimacy of algorithmic systems because they have limited voice, explanation, or control over the rules that shape their platform participation (Binns et al., 2018; Bucher, 2012; Shin, 2020).

Algorithmic technicality may also weaken users’ fairness evaluations when data-driven prediction is perceived as excessive, reductive, or insufficiently transparent. Although behavioral data can improve recommendation efficiency, brief views, accidental clicks, or temporary interests may be captured and transformed into future recommendations (Adomavicius & Tuzhilin, 2005). Users may then question whether algorithms genuinely understand their preferences, whether past behavior is overused to define them, and whether personal data are used to shape their information environment in ways that are difficult to explain or contest (Deldjoo et al., 2024; Gillespie, 2014; Shin & Park, 2019).

Fairness evaluations provide a cognitive basis for algorithmic resistance. When users perceive algorithmic systems as fair, consistent, and unbiased, they are more likely to regard algorithmic intervention as legitimate and acceptable. By contrast, when users perceive algorithmic bias, inconsistent rules, unreasonable data use, or insufficient explanation, algorithmic legitimacy may be weakened. In such cases, resistance can be understood as a legitimacy-based response to algorithmic influence that users perceive as unfair or insufficiently justified. Users may ignore, avoid, question, or bypass algorithms not simply because recommendations are unsatisfactory, but because the algorithmic arrangements behind them are perceived as illegitimate (Binns et al., 2018; Kim & Kankanhalli, 2009; Velkova & Kaun, 2021). Taken together, algorithmic power and algorithmic technicality may weaken users’ fairness evaluations, and weakened fairness evaluations may in turn provide a cognitive pathway through which perceived algorithmic characteristics are associated with stronger resistance-related behaviors. Accordingly, this study proposes:

**H1a.**  *Fairness evaluations mediate the relationship between algorithmic power and algorithmic resistance, such that algorithmic power is associated with stronger algorithmic resistance through lower fairness evaluations.*

**H1b.**  *Fairness evaluations mediate the relationship between algorithmic technicality and algorithmic resistance, such that algorithmic technicality is associated with stronger algorithmic resistance through lower fairness evaluations.*

### 2.4. The Mediating Role of Negative Emotions

The same perceived characteristics of platform algorithms may also be associated with algorithmic resistance through users’ negative emotions. Whereas fairness evaluations represent a cognitive pathway, negative emotions capture users’ affective discomfort when algorithmic systems are experienced as intrusive, repetitive, restrictive, or difficult to control. Negative emotions refer to users’ feelings of annoyance, frustration, tension, discomfort, or perceived intrusion during interactions with platform algorithms (Watson et al., 1988). Unlike fairness evaluations, negative emotions do not necessarily arise from explicit judgments about algorithmic rules; they may also emerge from accumulated platform experiences in which recommendations feel mismatched, repetitive, intrusive, or difficult to avoid (Bucher, 2017; Yang et al., 2024). Prior research further suggests that such affective responses shape how users cope with or continue using technological systems when they experience them as intrusive or difficult to control (Beaudry & Pinsonneault, 2010; Lee, 2018).

Algorithmic power may intensify users’ negative emotions. Psychological reactance theory suggests that individuals tend to experience dissatisfaction, resentment, or anger when they perceive their freedom of choice as being restricted by an external force (Brehm, 1966). In platform environments, algorithms continuously influence what users see, where they spend time, and how they interact with content through mechanisms of algorithmic control and behavioral guidance (Alizadeh et al., 2023; Kellogg et al., 2020). When users feel pushed by algorithms, trapped in repetitive content categories, or unable to escape platform-defined recommendation logics, algorithmic power may be experienced as irritation, fatigue, or a sense of being manipulated (Yang et al., 2024). Such affective reactions are especially likely when users perceive limited room for adjustment, refusal, or meaningful control.

Algorithmic technicality may also become emotionally consequential. Although data-driven prediction can improve recommendation relevance, it may become unsettling when users feel continuously observed, categorized, and anticipated (Kang et al., 2026). Precision in recommendation can be useful in some situations, but it may become intrusive when platforms repeatedly recommend content users do not genuinely need, reinforce interest labels that users are trying to escape, or appear to “know” users without genuinely “understanding” them (Yang et al., 2024). These experiences resonate with prior findings that algorithmic decisions can evoke emotional responses when users perceive them as intrusive, unfair, or difficult to contest (Lee, 2018). Thus, algorithmic technicality may generate negative emotions when data-driven prediction is experienced as excessive, invasive, or insufficiently controllable.

Negative emotions provide an affective basis for algorithmic resistance. When users repeatedly experience annoyance, frustration, tension, or a sense of being manipulated during platform use, resistance-related behaviors may become ways to relieve emotional strain, reduce unwanted algorithmic intervention, and regain a sense of autonomy. Users may ignore recommendations, avoid content, reduce interaction, or change usage patterns not only because they judge algorithmic systems as illegitimate, but also because algorithmic experiences become emotionally unpleasant (Beaudry & Pinsonneault, 2010; Jung & Park, 2018; Kim & Kankanhalli, 2009). Taken together, algorithmic power and algorithmic technicality may intensify users’ negative emotions, and stronger negative emotions may in turn provide an affective pathway through which perceived algorithmic characteristics are associated with stronger resistance-related behaviors. Accordingly, this study proposes:

**H2a.**  *Negative emotions mediate the relationship between algorithmic power and algorithmic resistance, such that algorithmic power is associated with stronger algorithmic resistance through stronger negative emotions.*

**H2b.**  *Negative emotions mediate the relationship between algorithmic technicality and algorithmic resistance, such that algorithmic technicality is associated with stronger algorithmic resistance through stronger negative emotions.*

**Figure 1:**
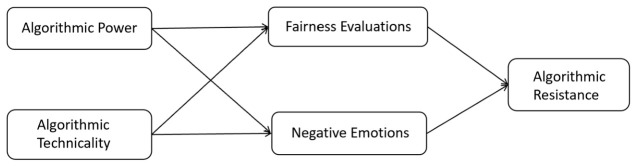
Conceptual model.

The proposed conceptual model is shown in Figure 1.

## 3. Materials and Methods

### 3.1. Sampling and Research Procedure

To empirically test the proposed model, this study employed a quantitative research design based on an online survey. Data were collected through multi-channel online recruitment, including online questionnaire platforms, social media sharing, and university-based online communities. The target respondents were algorithm-experienced users, defined as individuals who had used social media apps with algorithmic functions and reported their duration of use in the questionnaire.

The questionnaire included measures of the focal constructs, use duration of algorithm-driven social media apps, and demographic characteristics. All responses were collected anonymously, and an informed consent statement was presented at the beginning of the survey to inform participants of the study purpose, data use, confidentiality protection, and their right to withdraw at any time.

Before the formal survey, a pilot test was conducted to assess the clarity and reliability of the measurement items, and the results indicated satisfactory internal consistency across all constructs (Nunnally, 1978). The formal data collection was conducted between October and November 2025. The online questionnaire included attention-check and logic-check items, and additional screening was conducted based on completion time, patterned answering, and response consistency. The exported dataset contained 585 completed questionnaires. After excluding 20 invalid responses, 565 valid questionnaires were retained for analysis, yielding a valid response rate of 96.6% among completed questionnaires. The final sample consisted of algorithm-experienced social media users, with demographic characteristics and platform-use duration reported in Table 1.

### 3.2. Measures

All constructs were measured using multi-item scales adapted from prior literature and modified to fit the context of algorithm-driven social media platforms. Because most original scales were developed in English, Brislin’s (1980) back-translation procedure was employed to ensure semantic equivalence. The items were first translated into Chinese and then independently back-translated into English by bilingual experts. Discrepancies were discussed and revised until semantic consistency was achieved. Except for demographic variables, all items were measured using five-point Likert scales, ranging from 1 = strongly disagree to 5 = strongly agree. The full measurement items are reported in Appendix A.

Algorithmic power and algorithmic technicality were measured as two perceived characteristics of platform algorithms. Algorithmic power was measured with five items adapted from prior research on perceived algorithmic control and algorithmic management (Alizadeh et al., 2023; Kellogg et al., 2020). The items were revised to capture users’ perceptions of algorithmic rewards, penalties, visibility allocation, and behavioral constraints in platform environments. Algorithmic technicality was measured with three items adapted from research on recommender systems and algorithmic decision-making (Adomavicius & Tuzhilin, 2005). These items captured users’ perceptions of behavioral data use, preference inference, and personalized recommendations.

Fairness evaluations were measured with seven items. The scale was adapted from established measures of organizational justice (Colquitt, 2001) and further revised to fit algorithmic decision-making contexts based on prior research on algorithmic fairness (Binns et al., 2018). The items assessed whether users perceived algorithmic decision-making processes as fair, consistent, and free from bias. Negative emotions were measured with six items. The scale was adapted from established measures of negative effect (Watson et al., 1988) and prior research on emotional responses to information technology (Beaudry & Pinsonneault, 2010). The items captured feelings such as frustration, irritation, discomfort, and perceived intrusion in response to algorithmic recommendations. Algorithmic resistance was measured with seven items. The scale was adapted from established measures of user resistance to information systems (Kim & Kankanhalli, 2009) and prior research on everyday resistance practices in algorithmic environments (Karizat et al., 2021). The items captured behaviors such as ignoring, questioning, avoiding, and circumventing algorithmic recommendations.

### 3.3. Data Analysis

Data were analyzed using IBM SPSS Statistics 26.0 and IBM SPSS AMOS 26.0 (IBM Corp., Armonk, NY, USA). SPSS was used to examine descriptive statistics, reliability coefficients, and preliminary data quality, while AMOS was used to conduct confirmatory factor analysis (CFA), structural equation modeling (SEM), and bootstrapping-based mediation tests.

The measurement model was evaluated using commonly reported fit indices, including χ^2^/df, IFI, CFI, TLI, and RMSEA. Convergent validity was assessed based on standardized factor loadings, composite reliability (CR), and average variance extracted (AVE), and discriminant validity was evaluated using the Fornell–Larcker criterion. Common method bias was examined by comparing the hypothesized measurement model with a CFA model including a common method factor.

After confirming the adequacy of the measurement model, the hypothesized mediation model was tested using SEM. Gender, age, and education were included as control variables for the endogenous variables, including fairness evaluations, negative emotions, and algorithmic resistance. Finally, the mediating effects of fairness evaluations and negative emotions were examined using 5000 bootstrap resamples. Mediation effects were considered significant when the 95% confidence intervals did not include zero.

## 4. Results

### 4.1. Measurement Model

To assess the reliability and validity of the measurement model, confirmatory factor analysis (CFA) was conducted using AMOS 26.0. The hypothesized five-factor model, including algorithmic power, algorithmic technicality, fairness evaluations, negative emotions, and algorithmic resistance, showed a good fit to the data: χ^2^ = 493.309, df = 340, χ^2^/df = 1.451, IFI = 0.97, CFI = 0.97, TLI = 0.96, and RMSEA = 0.032. These indices indicated an acceptable fit of the measurement model (Bagozzi & Yi, 1988; Hu & Bentler, 1999).

Reliability and convergent validity were assessed based on standardized factor loadings, Cronbach’s α, composite reliability (CR), and average variance extracted (AVE). As shown in Table 2, the standardized factor loadings ranged from 0.667 to 0.844, exceeding the recommended threshold of 0.60. Cronbach’s α values ranged from 0.855 to 0.904, and CR values ranged from 0.855 to 0.905, both above the recommended threshold of 0.70, indicating satisfactory internal consistency and construct reliability. AVE values ranged from 0.548 to 0.663, exceeding the recommended threshold of 0.50, thereby supporting acceptable convergent validity (Fornell & Larcker, 1981).

Discriminant validity was evaluated using the Fornell–Larcker criterion. As shown in Table 3, the square roots of AVE for each construct were greater than the corresponding inter-construct correlations, indicating adequate discriminant validity. The correlation results were also consistent with the expected directions among the focal constructs.

To examine the potential impact of common method bias, Harman’s single-factor test was conducted as a preliminary diagnostic procedure (Podsakoff et al., 2003). The results showed that the first unrotated factor accounted for 33.93% of the total variance, which was below the commonly used benchmark of 50%, suggesting that no single factor dominated the covariance among the measures. In addition, a CFA model including a common method factor was estimated (Malhotra et al., 2006). Compared with the hypothesized five-factor model, the inclusion of the common method factor resulted in only marginal improvements in model fit: χ^2^ = 456.614, df = 312, χ^2^/df = 1.463, IFI = 0.97, CFI = 0.97, TLI = 0.96, and RMSEA = 0.031. The changes in key fit indices were minimal, suggesting that common method bias was unlikely to substantially bias the results.

Taken together, these results provide evidence for the reliability, convergent validity, and discriminant validity of the measurement model, justifying its use for subsequent structural analysis.

### 4.2. Structural Model Testing

**Figure 2:**
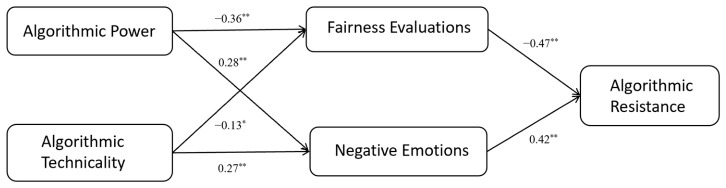
Structural model results. Note: * *p* < 0.05, ** *p* < 0.01.

To test the proposed structural model, structural equation modeling (SEM) was conducted using AMOS 26.0. Gender, age, and education were included as control variables in the structural model to account for potential demographic differences in users’ responses to platform algorithms. The structural model fit the data well: χ^2^ = 677.530, df = 449, χ^2^/df = 1.509, IFI = 0.96, CFI = 0.96, TLI = 0.95, and RMSEA = 0.035. The results of the structural model are presented in Figure 2.

The results show that algorithmic power was negatively associated with users’ fairness evaluations (β = −0.36, *p* < 0.01). Algorithmic technicality was also negatively associated with fairness evaluations (β = −0.13, *p* < 0.05). These findings indicate that fairness evaluations were lower when users perceived platform algorithms as more controlling or more data-driven. In addition, algorithmic power was positively associated with negative emotions (β = 0.28, *p* < 0.01), and algorithmic technicality was also positively associated with negative emotions (β = 0.27, *p* < 0.01). Thus, both perceived algorithmic control and data-driven predictive capacity were associated with stronger negative affective responses.

Regarding the associations between the mediators and algorithmic resistance, fairness evaluations were negatively associated with resistance (β = −0.47, *p* < 0.01), whereas negative emotions were positively associated with resistance (β = 0.42, *p* < 0.01). This pattern indicates that algorithmic resistance was associated with both lower perceived fairness and stronger emotional discomfort toward platform algorithms.

Overall, the structural model results were consistent with the proposed mediation logic. The estimated component paths provide the basis for testing the hypothesized indirect effects in the following section.

### 4.3. Mediation Effects

To examine the mediating roles of fairness evaluations and negative emotions, a bootstrapping procedure with 5000 resamples was conducted using AMOS. Direct paths from algorithmic power and algorithmic technicality to algorithmic resistance were also estimated to assess the remaining direct effects after accounting for the mediators. The results are presented in Table 4.

The indirect effect of algorithmic power on algorithmic resistance through fairness evaluations was significant (indirect effect = 0.146, 95% CI [0.098, 0.201]), supporting H1a. Because algorithmic power was negatively associated with fairness evaluations, and lower fairness evaluations were associated with stronger resistance, this pathway produced a positive indirect effect. The indirect effect of algorithmic technicality on algorithmic resistance through fairness evaluations was also significant (indirect effect = 0.036, 95% CI [0.002, 0.076]), supporting H1b. These results indicate that fairness evaluations mediated the associations between both perceived algorithmic characteristics and algorithmic resistance.

The indirect effect of algorithmic power on algorithmic resistance through negative emotions was significant (indirect effect = 0.091, 95% CI [0.056, 0.143]), supporting H2a. The indirect effect of algorithmic technicality on algorithmic resistance through negative emotions was also significant (indirect effect = 0.075, 95% CI [0.045, 0.115]), supporting H2b. These findings show that negative emotions also served as a mediating pathway linking perceived algorithmic characteristics to resistance-related behaviors.

The direct effects of algorithmic power (direct effect = 0.085, 95% CI [0.001, 0.175]) and algorithmic technicality (direct effect = 0.124, 95% CI [0.050, 0.196]) on algorithmic resistance remained significant after including the mediators, indicating partial mediation. Additional comparisons showed that the two indirect paths did not differ significantly because the confidence intervals included zero (algorithmic power: difference = 0.054, 95% CI [−0.001, 0.112]; algorithmic technicality: difference = −0.039, 95% CI [−0.079, 0.002]). This suggests that fairness evaluations and negative emotions exerted statistically comparable mediating effects.

Overall, the results support a dual-path mediation mechanism in which fairness evaluations and negative emotions explain how perceived algorithmic characteristics are associated with algorithmic resistance through cognitive and affective pathways.

## 5. Discussion

### 5.1. Main Findings

This study examined why users report resistance to platform algorithms in algorithm-driven social media environments. Moving beyond the dominant focus on algorithmic acceptance, trust, and continued use, it conceptualized algorithmic resistance as everyday behavioral responses shaped by users’ cognitive and affective experiences of algorithmic power and data-driven inference. The empirical results supported the proposed parallel mediation model: algorithmic power and algorithmic technicality were associated with algorithmic resistance through lower fairness evaluations and stronger negative emotions. Taken together, the findings suggest that algorithmic resistance is not simply a reaction to inaccurate or ineffective recommendations. Rather, it reflects users’ responses to two deeper experiences of algorithmic influence: being governed by platform systems and being inferred through data-driven prediction.

Perceived algorithmic power was associated with lower fairness evaluations, stronger negative emotions, and greater algorithmic resistance. This result is consistent with research that conceptualizes platform algorithms as systems that organize visibility, distribute opportunities, and shape the conditions of user action (Beer, 2017; Bucher, 2012; Gillespie, 2014). The finding suggests that algorithmic power becomes psychologically consequential when users perceive algorithms not merely as recommendation tools, but as governance systems that decide what becomes visible, rewarded, restricted, or actionable. Under such conditions, fairness concerns arise because users are subject to platform rules that appear asymmetrical, opaque, and difficult to contest. Emotional discomfort may also emerge because algorithmic control reduces users’ sense of autonomy within platform-defined visibility and recommendation logics. Thus, algorithmic power links platform governance to resistance by weakening users’ sense of fairness and increasing the felt burden of algorithmic control (Kellogg et al., 2020; Zhu et al., 2026).

Algorithmic technicality was also associated with lower fairness evaluations and stronger negative emotions. This finding shows that users’ responses to algorithms are not shaped only by overt control or restriction, but also by how they experience data-driven tracking, inference, and prediction. Research on recommender systems has shown that personalized recommendations shape information exposure and user judgment while raising questions about fairness and stakeholder value trade-offs (Deldjoo et al., 2024). The present findings add that technical sophistication itself may become a source of discomfort when users experience it as reductive or intrusive. Users may not resist personalization simply because it is inaccurate; they may resist the feeling of being continuously read, classified, and predicted through behavioral traces (Bucher, 2017; Karizat et al., 2021). A brief view, accidental click, or temporary interest may be translated into repeated recommendations, making users feel that the platform recognizes data patterns without genuinely understanding their preferences. In this sense, algorithmic technicality becomes a source of resistance not because it is technically weak, but because it can make users feel overly knowable, classifiable, and difficult to escape.

The mediating results further show that fairness evaluations and negative emotions represent distinct but complementary pathways. Prior work on algorithmic fairness and explainability has shown that users’ responses to algorithmic systems depend not only on outcomes but also on perceived transparency, consistency, and legitimacy of algorithmic procedures (Binns et al., 2018; Shin, 2020; Shin & Park, 2019). Consistent with this view, lower fairness evaluations indicate that resistance may function as a legitimacy-based response: users become less willing to comply with algorithmic arrangements when the rules, data practices, or recommendation outcomes appear unfair. Negative emotions were also associated with stronger resistance, echoing information systems research showing that affective responses matter when technological systems are experienced as intrusive, frustrating, or difficult to control (Beaudry & Pinsonneault, 2010; Kim & Kankanhalli, 2009). This affective pathway suggests that resistance may also operate as a coping response through which users seek to reduce emotional strain and regain a sense of autonomy. Fairness evaluations and negative emotions therefore do not duplicate each other; they capture different ways in which users translate algorithmic experience into resistance-related behavior.

At the same time, the parallel mediation model should not be interpreted as implying that fairness evaluations and negative emotions are psychologically isolated. In everyday platform experiences, perceptions of unfairness may coexist with, and potentially intensify, negative emotions such as frustration, irritation, and discomfort. The supplementary post hoc analysis showed that fairness evaluations were significantly associated with negative emotions (β = −0.32, *p* < 0.01), suggesting that the cognitive and affective pathways are analytically distinguishable but experientially interrelated. Taken together, these findings suggest that algorithmic resistance is neither a simple rejection of technology nor a purely emotional reaction to unwanted recommendations. Rather, it is an everyday form of user agency that emerges when platform algorithms are perceived as unfair systems of control and intrusive systems of data-driven inference (Bonini & Treré, 2024; Velkova & Kaun, 2021).

### 5.2. Theoretical Implications

This study contributes to platform algorithm research by shifting attention from algorithmic acceptance to algorithmic resistance as an important dimension of human–algorithm relations. While prior research has examined users’ trust, acceptance, sense-making, and everyday negotiation of algorithmic systems (Bishop, 2019; Bucher, 2017; Karizat et al., 2021; Shin, 2020; Velkova & Kaun, 2021), less attention has been paid to the psychological mechanisms through which perceived algorithmic characteristics become translated into resistance-related behaviors. By linking algorithmic power and algorithmic technicality to resistance through fairness evaluations and negative emotions, this study extends existing work from describing resistant practices to explaining the cognitive and affective conditions under which algorithmic resistance emerges.

The distinction between algorithmic power and algorithmic technicality further refines how platform algorithms are conceptualized in user research. Prior research has shown that algorithms organize visibility, structure participation, and shape platform power relations (Beer, 2017; Bucher, 2012; Gillespie, 2014; Kellogg et al., 2020), while recommender system research has emphasized data processing, preference inference, prediction, and personalization as core technical functions of algorithmic systems (Adomavicius & Tuzhilin, 2005; Deldjoo et al., 2024). By separating these two dimensions, this study avoids treating platform algorithms as a single undifferentiated source of influence. Algorithmic power captures the governance dimension of platform algorithms, whereas algorithmic technicality captures their data-driven inferential dimension. This distinction clarifies that users may respond not only to how algorithms allocate visibility, opportunities, and constraints, but also to how algorithms read, classify, and anticipate them through behavioral data. It therefore offers a more differentiated conceptual basis for understanding why platform algorithms may become objects of resistance.

This study further contributes by theorizing fairness evaluations and negative emotions as parallel cognitive and affective pathways of algorithmic resistance. Research on algorithmic fairness and explainability has shown that users’ responses to algorithmic systems depend on perceived transparency, consistency, legitimacy, and procedural fairness (Binns et al., 2018; Colquitt, 2001; Shin, 2020; Shin & Park, 2019), while information systems research has shown that users’ affective responses shape how they cope with intrusive, frustrating, or difficult-to-control technologies (Beaudry & Pinsonneault, 2010; Kim & Kankanhalli, 2009). By bringing these two perspectives together, this study shows that algorithmic resistance is better understood as emerging from both legitimacy-based judgments and affective discomfort. In doing so, it extends critical socio-technical perspectives on platform algorithms to the context of Chinese algorithm-driven social media platforms, where recommendation, ranking, traffic distribution, and commercialized content circulation are deeply embedded in everyday platform use. This contextual contribution provides an empirical basis for future cross-platform and cross-cultural comparisons of how users evaluate, experience, and resist algorithmic systems.

### 5.3. Practical Implications

The findings offer practical implications for platform designers and managers. Platforms should not treat user resistance to algorithms as a problem that can be solved simply by improving recommendation accuracy or engagement performance. More accurate personalization may still generate resistance when users experience it as excessive tracking, behavioral steering, or unwanted intervention. Recommendation systems should therefore be evaluated not only by clicks, retention, and engagement, but also by whether users perceive them as fair, adjustable, and respectful of autonomy. From this perspective, sustainable algorithmic governance requires platforms to balance personalization efficiency with procedural fairness, user control, and emotional well-being.

Platforms should also pay closer attention to users’ fairness evaluations and emotional experiences. Lower fairness evaluations were associated with stronger algorithmic resistance, suggesting that perceived inconsistency, bias, opacity, or lack of contestability may become important sources of user concern. To strengthen the perceived legitimacy of algorithmic governance, platforms could make recommendation logic more interpretable, clarify how user data are used, and provide users with more meaningful control over recommendation preferences. For users whose visibility, reach, or account access is affected by algorithmic rules, clearer explanations of traffic distribution, visibility allocation, penalties, and restrictions may help reduce perceptions of arbitrariness. Negative emotions were also associated with stronger resistance-related behaviors, suggesting that users may resist algorithms when they feel irritated, interrupted, misread, or manipulated. Platforms could therefore avoid overly repetitive or intrusive recommendations, improve the effectiveness of feedback tools, and make it easier for users to reset, pause, or adjust recommendation logic. These measures may help reduce both legitimacy concerns and emotional burden in users’ everyday encounters with platform algorithms.

### 5.4. Limitations and Future Research

This study has several limitations. First, the use of cross-sectional survey data limits the ability to establish causal order. Although the proposed model is theoretically grounded, the observed relationships should be interpreted as associations rather than definitive causal effects. Future research could use longitudinal designs, experiments, or platform behavioral data to examine how specific algorithmic experiences may lead to changes in fairness evaluations, negative emotions, and resistance-related behaviors over time. In addition, the sample was obtained through multi-channel online recruitment and should not be interpreted as statistically representative of all social media users. Because this study examined users’ general perceptions of algorithm-driven social media platforms, future research could use more diverse or stratified samples and compare different platforms, user roles, and cultural contexts to test whether the proposed mechanisms operate similarly across platform ecosystems.

Second, this study treated algorithmic resistance as an overall behavioral tendency and modeled fairness evaluations and negative emotions as parallel mediating mechanisms. This design was appropriate for examining the general psychological mechanism of algorithmic resistance, but it could not capture the full diversity of users’ resistance practices or the possible temporal relationship between cognitive and affective responses. Future research could develop more fine-grained measures of algorithmic resistance and examine whether fairness evaluations and negative emotions operate in serial, reciprocal, or dynamic ways. Moreover, users’ responses to platform algorithms may also be shaped by broader algorithm-related orientations, such as generalized skepticism toward algorithms, perceived autonomy loss, algorithmic imaginaries, or prior assumptions about platform systems. Future studies could therefore incorporate algorithmic literacy or algorithm awareness as potential antecedents or moderators to clarify how users interpret data-driven technicality, emotional discomfort, and resistance to platform algorithms (Dogruel et al., 2022; Gagrčin et al., 2026).

## 6. Conclusions

This study examined why users report resistance to platform algorithms in algorithm-driven social media environments. The findings suggest that algorithmic resistance is not merely a response to inaccurate or opaque recommendations but also reflects how users perceive algorithms as systems of platform control and data-driven inference. Algorithmic power and algorithmic technicality were associated with lower fairness evaluations and stronger negative emotions, which were further linked to resistance-related behaviors in everyday platform use. By identifying fairness evaluations and negative emotions as two parallel psychological mechanisms, this study offers a cognitive–affective account of algorithmic resistance. This perspective contributes to a more balanced understanding of human–algorithm relations, recognizing not only the efficiency and personalization benefits of algorithms, but also users’ efforts to negotiate autonomy under conditions of platform control and datafication.

## Figures and Tables

**Table 1:** Sample Characteristics (*N* = 565).

Attributes	Categories	Number	Percentage
Gender	Male	273	48.30
	Female	292	51.70
Age	18–30	287	50.80
	31–40	160	28.30
	41–50	77	13.60
	51 or over	41	7.30
Education	High school and below	98	17.30
	Junior college	116	20.50
	Bachelor	238	42.10
	Master or over	113	20.00
Usage duration	Less than 30 min	44	7.80
	30 min–1 h	89	15.80
	1–2 h	185	32.70
	2–3 h	146	25.80
	More than 3 h	101	17.90

**Table 2:** Results of Confirmatory Factor Analysis and Reliability Tests.

Variables	Item	Factor Loading	Cronbach’s α	CR	AVE
Algorithmic power	AP1	0.701	0.864	0.865	0.562
	AP2	0.758			
	AP3	0.748			
	AP4	0.819			
	AP5	0.716			
Algorithmic technicality	AT1	0.794	0.855	0.855	0.663
	AT2	0.818			
	AT3	0.831			
Fairness evaluations	FE1	0.667	0.893	0.894	0.548
	FE2	0.699			
	FE3	0.709			
	FE4	0.735			
	FE5	0.762			
	FE6	0.772			
	FE7	0.825			
Negative emotions	NE1	0.701	0.904	0.905	0.614
	NE2	0.740			
	NE3	0.782			
	NE4	0.800			
	NE5	0.825			
	NE6	0.844			
Algorithmic resistance	AR1	0.712	0.897	0.898	0.559
	AR2	0.713			
	AR3	0.686			
	AR4	0.716			
	AR5	0.708			
	AR6	0.841			
	AR7	0.840			

**Table 3:** Descriptive Statistics, Correlations, and Discriminant Validity.

	1	2	3	4	5	6	7	8
1. Gender								
2. Age	−0.056							
3. Education	−0.022	0.015						
4. Algorithmic power	0.016	0.024	0.086 *	**0.750**				
5. Algorithmic technicality	0.023	0.071	0.016	0.290 **	**0.814**			
6. Fairness evaluations	0.025	0.037	0.073	−0.323 **	−0.178 **	**0.740**		
7. Negative emotions	0.086 *	−0.022	−0.052	0.301 **	0.299 **	−0.389 **	**0.784**	
8. Algorithmic resistance	−0.025	0.039	−0.075	0.347 **	0.323 **	−0.547 **	0.522 **	**0.748**
Mean	1.520	1.770	2.650	3.216	3.282	2.904	3.141	3.103
SD	0.500	0.941	0.988	1.010	1.109	0.974	1.023	0.981

Note: *N* = 565. * *p* < 0.05, ** *p* < 0.01. Diagonal elements in bold represent the square roots of AVE, and off-diagonal elements represent correlations among variables.

**Table 4:** Results of Mediation Analysis (Bootstrapping).

Predictor	Path	Estimate	BootLLCI	BootULCI
Algorithmic power	Total effect	0.322	0.222	0.430
	Direct effect	0.085	0.001	0.175
	AP → FE → AR	0.146	0.098	0.201
	AP → NE → AR	0.091	0.056	0.143
Algorithmic technicality	Total effect	0.235	0.145	0.323
	Direct effect	0.124	0.050	0.196
	AT → FE → AR	0.036	0.002	0.076
	AT → NE → AR	0.075	0.045	0.115

Note: Bootstrapping was performed with 5000 resamples, and confidence intervals are bias-corrected. AP = Algorithmic power; AT = Algorithmic technicality; FE = Fairness evaluations; NE = Negative emotions; AR = Algorithmic resistance.

## Data Availability

The data presented in this study are available on reasonable request from the corresponding author due to privacy and ethical restrictions approved by the Ethics Review Committee of Xiamen University.

## Appendix A. Measurements

Algorithmic Power

AP1. The platform’s algorithm provides rewards (e.g., increased exposure or traffic support) based on my behavior.

AP2. The platform’s algorithm manages a large number of content creators and users.

AP3. The platform’s algorithm penalizes me for violations or inappropriate behavior.

AP4. The platform’s algorithm constrains how I use or create content on the platform.

AP5. The platform’s algorithm prevents me from using my account in the way I prefer.

Algorithmic Technicality

AT1. I believe the platform’s algorithm can accurately recommend content based on my preferences.

AT2. I understand that the platform’s recommendations are based on my browsing history and interaction data.

AT3. I am aware that the platform’s algorithm uses my personal data to provide personalized recommendations.

Fairness evaluations

FE1. The decisions made by the platform’s algorithm are fair.

FE2. The decisions made by the platform’s algorithm are just.

FE3. The platform’s algorithm evaluates all situations based on the same criteria.

FE4. The platform’s algorithm applies consistent evaluation standards to all users.

FE5. The platform’s algorithm discriminates against certain groups. (reverse-coded)

FE6. The platform’s algorithm favors certain groups. (reverse-coded)

FE7. The platform’s algorithm effectively avoids bias in its decision-making.

Negative Emotions

NE1. I feel annoyed when the recommended content does not match my interests.

NE2. I feel frustrated when the platform recommends content I am not interested in.

NE3. I feel hostility when the recommended content makes me feel disrespected.

NE4. I feel irritated when the platform’s recommendations are intrusive or disturbing.

NE5. I feel tense when the recommended content makes me feel stressed or uneasy.

NE6. I feel uncomfortable when the recommended content disrupts my concentration or makes me restless.

Algorithmic Resistance

AR1. I ignore notifications or prompts generated by the platform’s algorithm.

AR2. I pay little attention to content recommended by the platform’s algorithm.

AR3. I sometimes question the content recommended by the platform’s algorithm.

AR4. I try to avoid content recommended by the platform’s algorithm.

AR5. I bypass the platform’s algorithm when making choices.

AR6. I do not follow the behavior patterns suggested by the platform’s algorithm.

AR7. I take actions to avoid being monitored by the platform’s algorithm.
